# The Doctor’s Three Faces

**Published:** 1874-03

**Authors:** 


The Doctor's Three Faces.—Euricius Cordus, who died A.D 1535, doubtless gave the results of his own experience when he described the physician in these Latin verses:

"Tres medicus facies habiet: unam quando rogatur,
Angelicam; mox est, cum juvat, ipse Deus.
Post ubi curato poscit sua prœmia morbo,
Horridus apparat, terribilisque Sathan."

("Three faces wears the doctor; when first sought,
An angel's—and a God's, the cure half wrought;
But when, that cure complete, he seeks his fee,
The devil then looks less terrible than he.")

Pope sang in the same strain, although he was not one of the brotherhood:

"God and the doctor we alike adore,
But only when in danger—not before;
The danger o'er, both are alike requited,
God is forgotten, and the doctor slighted."

